# Turnover of Variant Surface Glycoprotein in Trypanosoma brucei Is a Bimodal Process

**DOI:** 10.1128/mBio.01725-21

**Published:** 2021-07-27

**Authors:** Paige Garrison, Umaer Khan, Michael Cipriano, Peter J. Bush, Jacquelyn McDonald, Aakash Sur, Peter J. Myler, Terry K. Smith, Stephen L. Hajduk, James D. Bangs

**Affiliations:** a Department of Microbiology and Immunology, Jacobs School of Medicine and Biomedical Sciences, University at Buffalo (SUNY), Buffalo, New York, USA; b Department of Biochemistry and Molecular Biology, University of Georgia, Athens, Georgia, USA; c South Campus Instrument Center, School of Dental Medicine, University at Buffalo (SUNY), Buffalo, New York, USA; d Center for Global Infectious Disease Research, Seattle Children’s Research Institute, Seattle, Washington, USA; e Department of Biomedical Informatics and Medical Education. University of Washington, Seattle, Washington, USA; f Department of Pediatrics. University of Washington, Seattle, Washington, USA; g Department of Global Health, University of Washington, Seattle, Washington, USA; h School of Biology, BSRC, University of St. Andrews, St Andrews, United Kingdom; i School of Chemistry, BSRC, University of St. Andrews, St Andrews, United Kingdom; Washington University School of Medicine

**Keywords:** trypanosome, variant surface glycoprotein, glycosylphosphatidylinositol, glycosylphosphatidylinositol-specific phospholipase C, extracellular vesicles

## Abstract

African trypanosomes utilize glycosylphosphatidylinositol (GPI)-anchored variant surface glycoprotein (VSG) to evade the host immune system. VSG turnover is thought to be mediated via cleavage of the GPI anchor by endogenous GPI-specific phospholipase C (GPI-PLC). However, GPI-PLC is topologically sequestered from VSG substrates in intact cells. Recently, A. J. Szempruch, S. E. Sykes, R. Kieft, L. Dennison, et al. (Cell 164:246–257, 2016, https://doi.org/10.1016/j.cell.2015.11.051) demonstrated the release of nanotubes that septate to form free VSG^+^ extracellular vesicles (EVs). Here, we evaluated the relative contributions of GPI hydrolysis and EV formation to VSG turnover in wild-type (WT) and GPI-PLC null cells. The turnover rate of VSG was consistent with prior measurements (half-life [*t*_1/2_] of ∼26 h) but dropped significantly in the absence of GPI-PLC (*t*_1/2_ of ∼36 h). Ectopic complementation restored normal turnover rates, confirming the role of GPI-PLC in turnover. However, physical characterization of shed VSG in WT cells indicated that at least 50% is released directly from cell membranes with intact GPI anchors. Shedding of EVs plays an insignificant role in total VSG turnover in both WT and null cells. In additional studies, GPI-PLC was found to have no role in biosynthetic and endocytic trafficking to the lysosome but did influence the rate of receptor-mediated endocytosis. These results indicate that VSG turnover is a bimodal process involving both direct shedding and GPI hydrolysis.

## INTRODUCTION

The parasitic protozoan Trypanosoma brucei (sp.) is endemic to sub-Saharan Africa, where it causes veterinary and human African trypanosomiasis. It has a digenetic life cycle that alternates between the tsetse fly vector (*Glossina*) and the mammalian host. The infectious metacyclic parasite is transmitted by bite to the mammalian host, where it replicates in the circulation and disseminates to skin and adipose tissue, ultimately invading the central nervous system (1–3). Each bloodstream form (BSF) cell is coated with a monolayer of a single variant surface glycoprotein (VSG), 10 million copies per cell, which shields underlying invariant membrane proteins from host immune responses. Trypanosomes avoid acquired immunity directed at VSG by a process of antigenic variation, where VSGs present on the cell surface are replaced with a newly transcribed *VSG*, thereby presenting a new antigen to the immune response (4, 5). There are up to 2,000 *VSG* genes per genome, and transcription is from 15 to 20 telomeric expression sites (ESs), only one of which is active at a time, thereby ensuring monoallelic exclusion. Antigenic variation occurs either by activating a new expression site with a different *VSG* gene or by gene conversion of the *VSG* in the active ES with a new gene from the genomic repertoire.

VSG comprises >90% of the cell surface proteome and is expressed at a high rate (∼30,000 molecules/min), producing enough homodimeric proteins for a new surface coat every 6-h cell cycle (5). Newly translated VSG is efficiently modified in the endoplasmic reticulum (ER) with glycosylphosphatidylinositol (GPI) anchors (6, 7) and then rapidly transported to the cell surface (half-life [*t*_1/2_] of ∼15 min) (8). The protein remains attached to the cell surface by its GPI anchor, two per homodimer. In normally replicating trypanosomes, VSG protein is very stable, with loss from the cell requiring multiple cell cycles (*t*_1/2_ of ∼30 h) (9, 10).

BSF cells have a GPI-specific phospholipase C (GPI-PLC) that is particularly enigmatic. Approximately 30,000 GPI-PLC molecules are associated with the cytoplasmic face of intracellular membranes (11–14), though the protein contains no clear N-terminal signal peptide or transmembrane domain. GPI-PLC is tightly regulated, possibly by dynamic acylation (15, 16), but when cells are disrupted by mechanical or hypotonic lysis, GPI-PLC gains full access to the cell surface and rapidly cleaves all the VSG GPI anchors (17, 18). Hydrolysis removes dimyristoylglycerol, releasing soluble VSG (sVSG) with the glycan portion of the GPI structure still attached to the C terminus. This moiety, the cross-reacting determinant (CRD), can be recognized by specific antibodies (19) and is diagnostic for GPI hydrolysis.

GPI-PLC’s major function is thought to be normal VSG turnover by regulated hydrolysis of cell surface GPI anchors, leading to its slow loss from the cell surface and accounting for normal turnover (9). However, its cytosolic membrane disposition presents a topological paradox, as VSG is located on the extracellular side of the plasma membrane of intact cells. GPI-PLC also reportedly stimulates the endocytosis of another VSG-related single-GPI-anchored protein, the transferrin receptor (TfR) (20). Other proposed functions for GPI-PLC include metabolism (21, 22), regulated release of VSG during differentiation to the procyclic insect stage (PCF) (23), and cleavage of misfolded GPI-anchored proteins prior to disposal by ER-associated degradation (ERAD) (24).

Extracellular vesicles (EVs) are produced by nearly every eukaryotic cell type and are vital for the pathogenesis of many disease-causing agents (25–27). They play roles in mediating cellular communication, altering immune reactions, influencing host gene expression, and spreading virulence factors and drug resistance markers between pathogens. EVs were recently described in T. brucei as extended nanotubes that bud from the membrane and septate to form free EVs of ∼80 nm (28). These EVs can interact with both host and pathogen by direct membrane fusion, receptor-mediated endocytosis, and receptor-mediated signal transduction. They are both VSG positive (VSG^+^) and GPI-PLC^+^, but it is not known to what extent they contribute to normal turnover of VSG. In this work, we investigated the relative contributions of GPI hydrolysis and EV shedding to total VSG turnover. Our results indicate that VSG-containing EVs make a very minor contribution to this process. Rather, molecular VSG is shed directly from the cell surface by GPI hydrolysis and, unexpectedly, by direct dissociation of VSG with fully intact GPI anchors.

## RESULTS

### Creation of a GPI-PLC null cell line.

**FIG 1:**
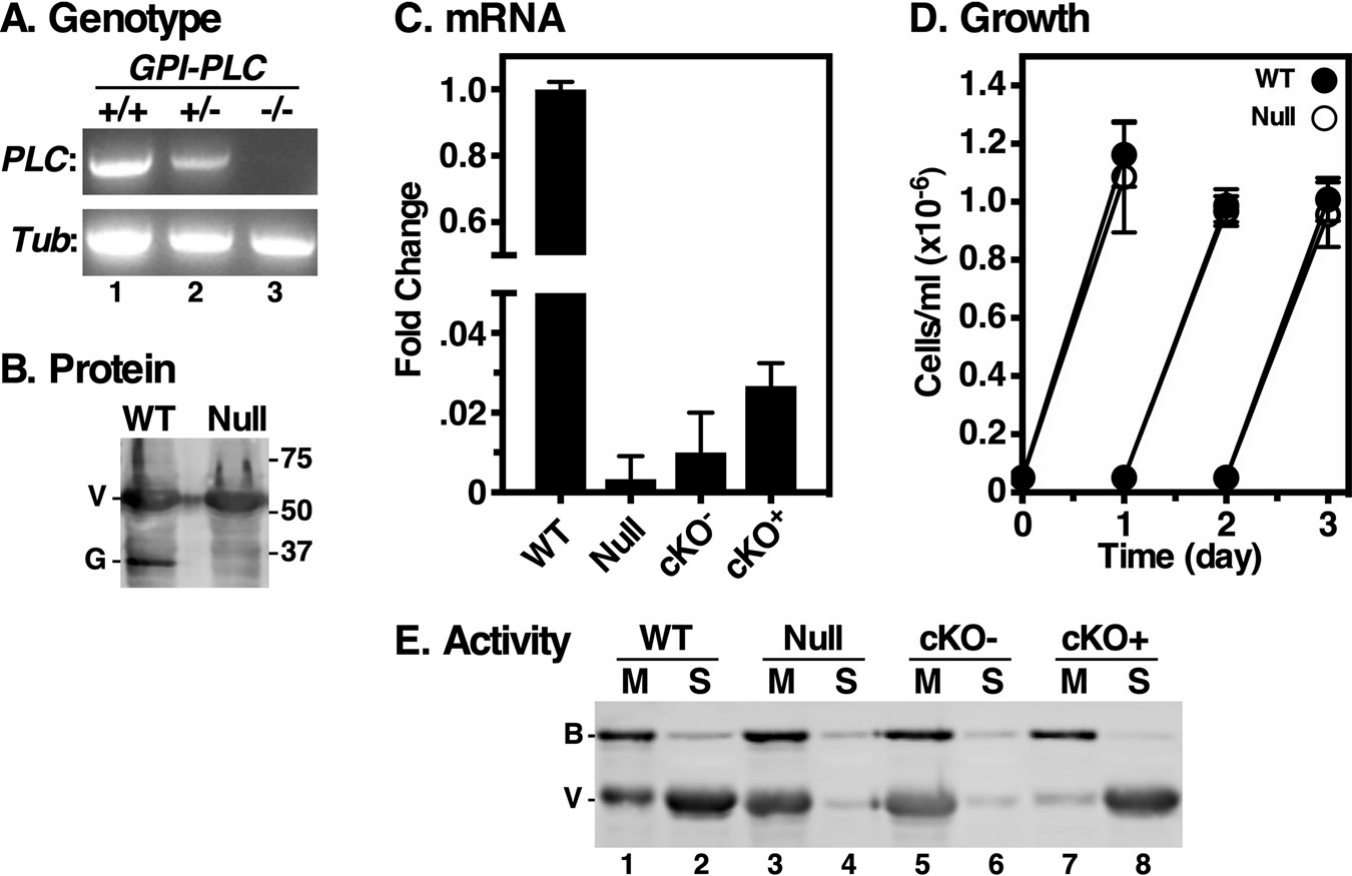
GPI-PLC gene knockout. (A) GPI-PLC wild type (+/+), single knockout (KO) (+/−) and double KO (−/−) cell lines were genotyped by PCR using specific primers for the GPI-PLC and tubulin gene open reading frames (ORFs). (B) GPI-PLC protein levels were detected by immunoblotting using anti-GPI-PLC (G) and anti-VSG (V; load control) antibodies. (C) qRT-PCR of WT, Null, cKO^−^ (Tet^−^), and cKO^+^ (Tet^+^) cell lines. Values are normalized to WT as fold change (means ± standard deviations [SDs], *n* = 3). (D) Growth curves of WT and GPI-PLC null cells, (means ± SDs, *n* = 3). Cells were seeded at 5 × 10^4^/ml and readjusted to starting density every 24 h. (E) Hypotonic lysates were prepared to activate endogenous GPI-PLC. Membrane (M) and supernatant (S) fractions were prepared and analyzed by immunoblotting using anti-VSG (V; endogenous GPI-PLC substrate) and anti-BIP (B; load control) antibodies (2 × 10^6^ cell equivalents per lane).

A cell line with a puromycin resistance cassette replacing a single GPI-PLC gene allele was created (GPI-PLC*^+/−^*). However, despite multiple subsequent attempts, we were unable to create a double knockout with a matched hygromycin resistance cassette. To circumvent this, we introduced a conditional GPI-PLC gene expression construct into the GPI-PLC*^+/−^* cell line under phleomycin selection and then targeted the second allele while inducing expression with tetracycline. This approach worked, but phleomycin was omitted from the clonal selection and, unexpectedly, the resultant clones had lost the inducible copy of the gene, as confirmed by PCR (Fig. 1A) (wild type [WT] versus +/− versus −/−). This “true” GPI-PLC^−/−^ null cell line retained puromycin and hygromycin resistance but was now phleomycin sensitive. Interestingly, loss of the inducible construct is similar to what occurred during the creation of the widely used GPI-PLC null cell line, RUMP528 (29). Nevertheless, because of the “arcane” derivation of the null cell line, we confirmed the discrete loss of both alleles by whole-genome sequencing (see Fig. S1 in the supplemental material).

10.1128/mBio.01725-21.1 FIG S1 Whole-genome sequencing of GPI-PLC knockout cell lines. Genomic DNA (gDNA) from the parental wild-type (WT; +/+), sKO-AddBack (+/−/+) and dKO (−/−) cell lines was used to prepare Illumina libraries using the NEB ultra II whole genome kit with 25 ng input gDNA and 5 cycles of PCR amplification. The libraries were sequenced using a PE75 protocol (75-bp paired-end reads) on a HiSeq 2000 at the UW Sequencing Northwest Genomics Center. The Geneious assembler (with medium-low sensitivity/fast parameters and 5 iterations) was used to align reads against the 317 contigs in the Tb427_2018 genome (TriTrypDB release 47) as well as the sequence of the four constructs used to create the cell lines. The figure shows screen captures (from Geneious) of read density for the GPI-PLC gene locus, with the reads per kilobase per million (RPKM) values for each gene shown for all three cell lines. The RPKM value for the GPI-PLC gene in WT also includes reads that mapped to the pLEW100X_GPI-PLC sequence, while the two values are shown separately for sKO-AddBack. Comparison of the RPKM values with the median value for all (diploid) genes in the Tb427_2018 genome indicates that there are two (haploid) copies of the GPI-PLC gene (both on Chr2) in the WT (+/+) cell line, two copies (one on Chr2 and one in the integrated pLEW100X_GPI-PLC sequence) in the sKO-AddBack (+/−/+) cell line, and a complete loss of all GPI-PLC gene sequences in the dKO (−/−) cell line. (means ± SDs, *n* = 3). Download FIG S1, TIF file, 2.9 MB. Copyright © 2021 Garrison et al. 2021 Garrison et al. https://creativecommons.org/licenses/by/4.0/ This content is distributed under the terms of the Creative Commons Attribution 4.0 International license.

Null cells completely lack GPI-PLC protein (Fig. 1B) and mRNA (Fig. 1C). Furthermore, growth of null parasites was unchanged from that of the wild type (WT) (Fig. 1D), confirming results previously seen with RUMP528. To confirm the loss of GPI-PLC activity in our null cells, we utilized the hypotonic release assay (18). WT and null cells were subjected to GPI-PLC-activating hypotonic lysis to allow cleavage of GPI anchors, thereby releasing soluble VSG from the cell surface. Crude membrane and supernatant fractions were then immunoblotted with anti-VSG antibody. WT cells showed normal GPI hydrolysis and release of surface VSG into the supernatant (Fig. 1E, lanes 1 and 2); the small remaining signal in the membrane fraction represents VSG located in intracellular (secretory and endocytic) compartments that is inaccessible to GPI-PLC during hypotonic lysis. Null cells, however, completely lacked GPI-PLC activity, as essentially all VSG remained membrane-associated, as did the control ER marker protein, BiP (Fig. 1E, lanes 3 and 4). To confirm that the lack of release was due to loss of GPI-PLC, we reintroduced the inducible copy of the GPI-PLC gene into the null cells, generating a conditional knockout (cKO) cell line. Quantitative reverse transcription-PCR (qRT-PCR) analysis indicated tetracycline-responsive expression of GPI-PLC, albeit at much lower levels than in wild-type cells (Fig. 1C). Nevertheless, this low level of expression was sufficient to fully restore release of sVSG during hypotonic lysis (Fig. 1E, compare lanes 5 and 6 versus 7 and 8).

### VSG turnover.

**FIG 2:**
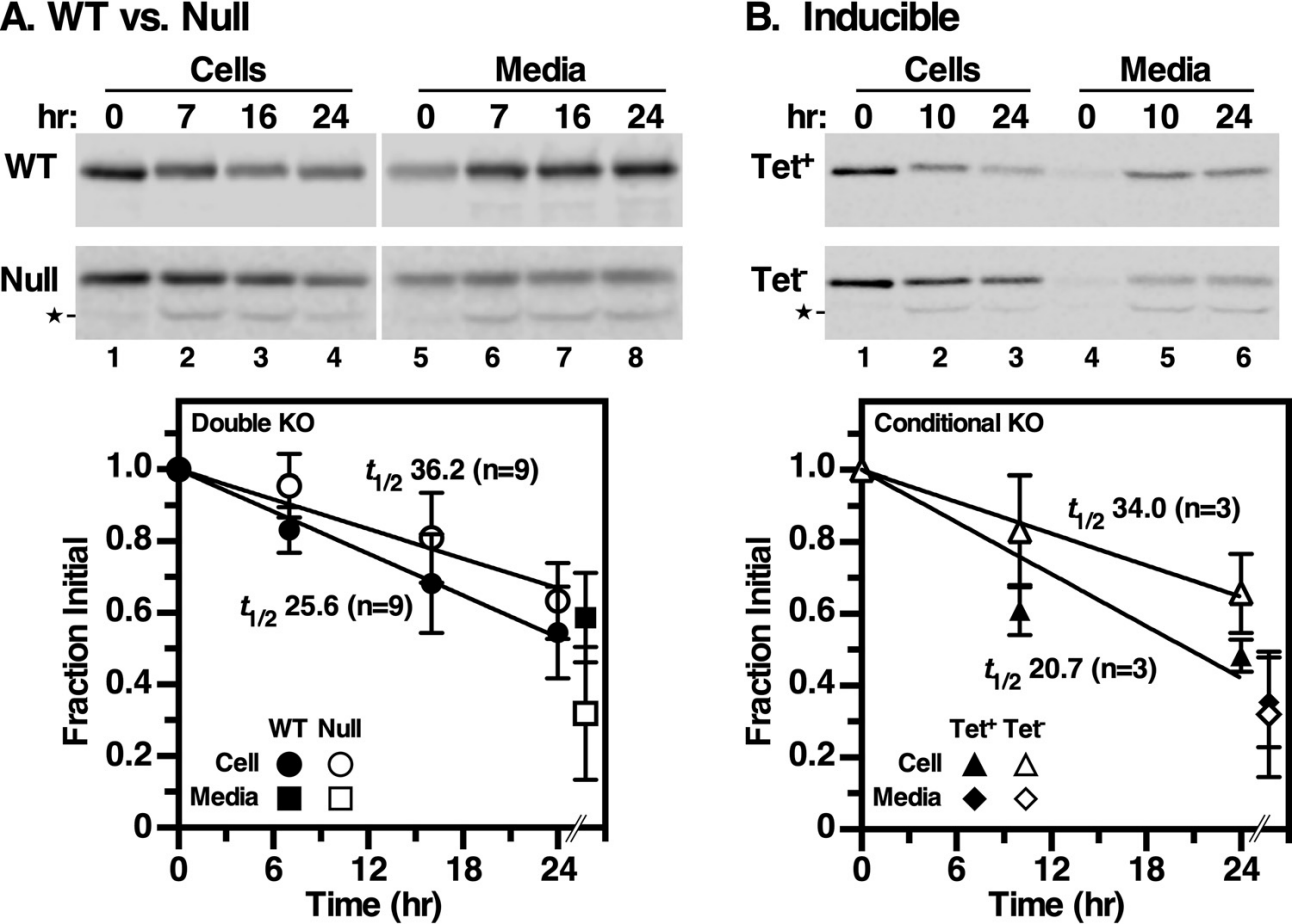
Turnover of VSG. Wild type versus null (*n* = 9) (A) and induced (Tet^+^) versus uninduced (Tet^−^) cKO (*n* = 3) (B) cell lines were analyzed by [^35^S]Met/Cys pulse-chase radiolabeling, and VSG was immunoprecipitated from cell and medium fractions at the indicated times. Precipitates were fractionated by SDS-PAGE and quantified by phosphorimaging (means ± SDs). Only the 24-h medium time point was quantified, and this is offset in the graphs for clarity of presentation; the *T*_0_ medium time point was subtracted to zero the data. A proteolytic fragment (star) seen only under GPI-PLC deficiency (null and Tet^−^ cKO) was included in the quantification for these experiments. Half-life times (*t*_1/2_; determined by linear regression) are indicated. *P* value was <0.001 for each set of GPI-PLC replete versus deficient cell lines.

We next assessed the rate of VSG turnover by pulse-chase radiolabeling in wild-type versus null cells. Normal VSG turnover occurred with a half time (*t*_1/2_) of ∼26 h (Fig. 2A), which is faster but still consistent with previous reports that were performed under somewhat different conditions (*t*_1/2_ of ∼32 h) (9, 10). However, VSG turnover was slower in null cells (Fig. 2A) (*t*_1/2_ of ∼36 h), with much less VSG appearing in the medium over the chase period. Noticeable in both the null cell and medium fractions was the time-dependent appearance of a smaller truncated VSG species that was absent in the comparable wild-type fractions (Fig. 2A, lanes 2 to 4 and 6 to 8). The relative size of this species is similar to that of a fragment previously seen during differentiation to the procyclic form of the parasite, which involves endoproteolytic cleavage and release of VSG by the zinc metalloprotease MSP-B (9, 30). Possibly, the loss of GPI-PLC leads to dysregulation of this enzyme. To confirm that both phenotypes—delayed turnover and truncation of VSG—result from GPI-PLC deficiency, we used the inducible cKO cell line. When GPI-PLC expression was induced (with tetracycline [Tet^+^]), a turnover rate similar to that in wild-type cells was observed (Fig. 2B) (*t*_1/2_ of ∼21 h versus 26 h), with no appearance of truncated VSG. However, in the absence of GPI-PLC expression (Tet^−^) VSG turnover dropped to the same rate as that for bona fide null cells (Fig. 2B) (*t*_1/2_ of ∼36 h versus 34 h). Strikingly, truncated VSG was again observed in both cell and medium fractions (Fig. 2B, bottom, lanes 2 and 3 and lanes 5 and 6). These results indicate that both phenotypes are due to GPI-PLC deficiency. All attempts to inhibit VSG truncation in null cells were either unsuccessful (FMK024, a T. brucei cathepsin L [*Tb*Cathepsin L] inhibitor) or inconclusive (specific metalloprotease inhibitors [structures SB227961 and SB227962] found in reference 31). Overall, these data confirm that VSG turnover occurs by shedding from the cell surface as originally reported (9, 10), with a basal rate of *t*_1/2_ of ∼26 h, somewhat faster than, but still similar to, the prior publications. However, GPI-PLC activity is apparently not required for VSG turnover, although its deficiency does delay shedding (*t*_1/2_ of ∼36 h).

### VSG shedding.

**FIG 3:**
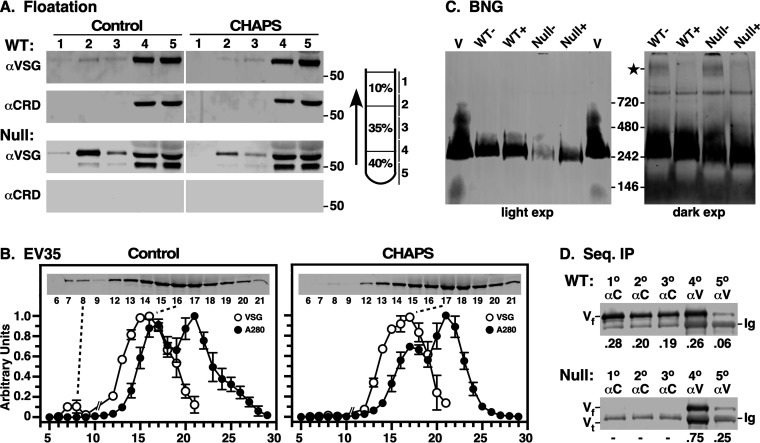
Characterization of shed VSG. Conditioned culture supernatants (CMs) were generated by incubating freshly harvested late-log-phase cells in fresh HMI-9 medium (6 h, 10^7^/ml). (A) Wild-type (WT) and GPI-PLC^−/−^ (null) conditioned culture supernatants (10^6^ cell equivalents) were fractionated by density floatation (diagram) in the absence and presence of 16 mM CHAPS (2× CMC). Gradient fractions were analyzed by immunoblotting with anti-VSG and anti-CRD antibodies. Vertical white lines indicate sections that were digitally excised for presentation after image processing. Mobility of molecular mass markers is indicated (right). WT versus null data are from different gels/images, and can only be compared qualitatively. (B) Gel filtration of WT conditioned medium. CM (0.5 ml) was gravity applied to an EV35 column, and 0.5-ml fractions were collected from *T*_0_. The *A*_280_ of each fraction was determined, and VSG was assayed by immunoblotting (inset). Note that fractions 10 and 11 were omitted. Each run was normalized to the fraction with the highest value, and data are presented as means ± SDs (*n* = 3). For CHAPS treatment, load samples were adjusted to 16 mM CHAPS and run in buffer with 1 mM CHAPS. (C) CM plus (+) or minus (−) 1% dodecylmaltoside (DDM) was fractionated by blue native gel electrophoresis and analyzed by immunoblotting. Purified sVSG (V; MITat1.2) was run as a control. A darker exposure of the CM lanes is presented on the right. DDM-sensitive high-molecular-weight VSG is indicated (star). Note that native VSG consistently migrates at approximately twice its known size (∼110 kDa) relative to the manufacturer supplied markers. This effect, which is likely due to the highly elongated and glycosylated nature of VSGs in general, has been noted by others (46). (D) WT and null CMs were subjected to sequential immunoprecipitation, 3× with anti-CRD (1°, 2°, and 3°) and then twice with anti-VSG (4° and 5°) antibodies. Precipitates were analyzed by immunoblotting with anti-VSG antibody. Mobilities of full-length (V_f_) and truncated (V_t_) VSG and background immunoglobulin heavy chain (Ig) are indicated. Fractional recoveries for each precipitation are shown (bottom).

To investigate the mode of VSG shedding, we subjected conditioned medium (CM) from cultures of WT and GPI-PLC^−/−^ null cell lines to density floatation, followed by immunoblotting with anti-VSG antibody to identify total protein and anti-CRD antibody to assess GPI hydrolysis (Fig. 3A). Detergent {16 mM 3-[(3-cholamidopropyl)-dimethylammonio]-1-propanesulfonate (CHAPS)} was included in control flotations to solubilize any VSG that might be in EVs. In WT cells, most VSG was found in the nonbuoyant fractions (4 and 5), and this was strongly reactive with the anti-CRD antibody, indicating hydrolyzed GPI anchors. A small amount of buoyant VSG was detected (fractions 2 and 3), but this was nonreactive with the anti-CRD antibody and was reduced by CHAPS treatment, consistent with shed EVs containing VSG with intact GPI anchors. In CM from null cells, the amount of nonbuoyant VSG (fractions 4 and 5) was greatly reduced relative to that of buoyant VSG (fractions 2 and 3), and all VSG was CRD negative. (Note that the relative total VSG signals in WT versus null cells cannot be compared directly, as these are different gels with different phosphorimages. For quantitative comparison see Fig. 3C.) Again, buoyant VSG was susceptible to CHAPS treatment. Interestingly, as seen in pulse-chase analyses (Fig. 2), both full-length and truncated VSG were found in the nonbuoyant fractions. We interpret this as heterodimeric VSG in which one subunit has been cleaved toward the C terminus, thereby removing one GPI anchor. As one BSF GPI anchor (dimyristoylglycerol) is not sufficient to maintain membrane association (32), these heterodimers are shed into the medium during culture and do not float during centrifugation. The results of gel filtration and blue native gel electrophoresis support this conclusion (see below).

Three additional physical assessments of shed VSG were performed. First, CM (WT only) was size selected on qEV35 columns (fractionation range 35 to 350 nm) (Fig. 3B, left). The *A*_280_ profile shows a leading peak (fraction number [fr. no.] 17) followed by a main peak that represents maximum inclusion of free serum proteins (fr. no. 21). Low levels of VSG were consistently found in the breakthrough fractions (fr. no. 7 and 8), but the overwhelming amount of VSG was centered on fractions 15 and 16, in advance of both the *A*_280_ peaks. Treatment with CHAPS (Fig. 3B, right) had minimal effect on elution of the main VSG peak but completely eliminated the small breakthrough VSG peak, suggesting this represents EVs equivalent to the buoyant material in floatation assays. Proteomic analysis of the main peak only detected VSG protein, and no other T. brucei proteins were detected (data not shown). Gel filtration of purified sVSG (∼110 kDa) indicated that it elutes identically to the main VSG peak in CM (see Fig. S2, fr. no. 16). Second, WT and Null CMs were fractionated by blue native gel (BNG) electrophoresis (Fig. 3C). In each case, VSG in CM overwhelming comigrated with the purified homodimeric sVSG control and was unaffected by addition of detergent. As expected, the overall signal was reduced in null CM relative to that from the WT. However, with overexposure, a small amount of detergent-sensitive high-molecular-weight VSG was detected, again consistent with the presence of a small amount of VSG in shed EVs. This material was somewhat more abundant in null cells, suggesting that the loss of GPI-PLC might influence EV production (see below). Finally, CM was subjected to sequential immunoprecipitation with anti-CRD and anti-VSG antibodies (Fig. 3D). Three rounds of treatment with anti-CRD antibodies were sufficient to deplete VSG with hydrolyzed GPI anchors from WT CM. Two additional rounds of treatment with anti-VSG antibodies precipitated the remaining VSG with intact GPI anchors, which represents approximately half of all VSG shed from intact cells (47.1% ± 14.0%, *n* = 3). As expected, no VSG was detected in null CM with the anti-CRD antibody, indicating that all shed VSG had intact GPI anchors. Collectively, these results indicate that most VSG is shed from WT cells as intact homodimers, which are quite evenly split between hydrolyzed and intact GPI anchors. The latter population accounts for all VSG turnover in GPI-PLC^−/−^ cells.

10.1128/mBio.01725-21.2 FIG S2 Calibration of EV35 columns. Thryoglobulin (Thyro; 660 kDa) and sVSG (∼110 kDa) were run on EV35 columns as for Fig. 3. Thryroglobulin was monitored by *A*_280_, and VSG was assayed by immunoblotting (inset). Note that fractions 10 and 11 were omitted. Each run was normalized to the fraction with the highest value. Download FIG S2, TIF file, 0.1 MB. Copyright © 2021 Garrison et al. 2021 Garrison et al. https://creativecommons.org/licenses/by/4.0/ This content is distributed under the terms of the Creative Commons Attribution 4.0 International license.

### EV formation.

**FIG 4:**
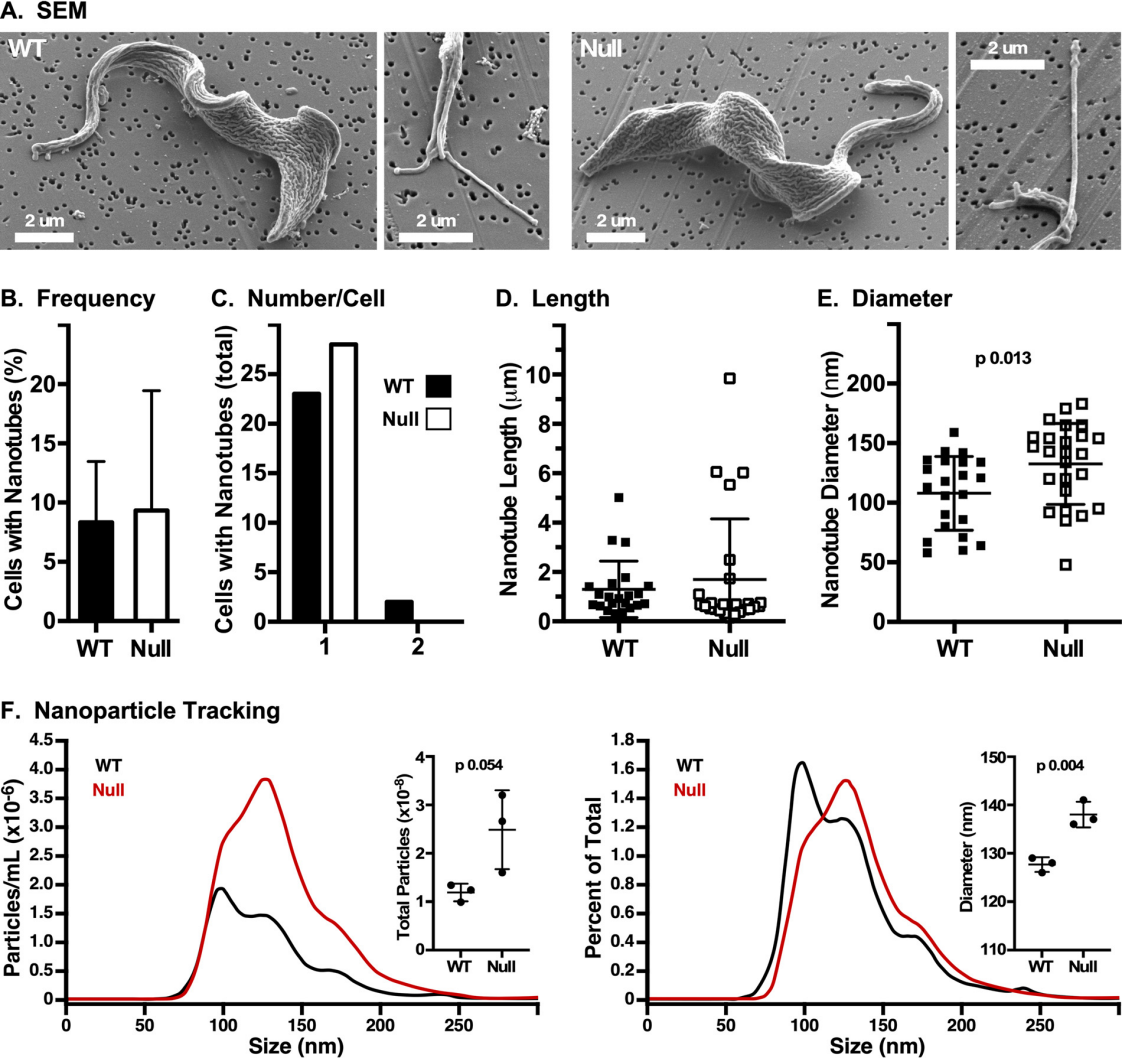
Formation of membrane nanotubes. Wild-type (WT) and GPI-PLC^−/−^ (null) cells were fixed, captured on 0.2-μm filters, and processed for SEM. (A) Representative SEM images of WT and null cells and cells with budding nanotubes. Nanotubes in WT and null cells were quantified for frequency (B), number per cell (C), length (D), and diameter (E) (*n* = 3, 100 cells per biological replicate). The statistical significance of WT versus null diameter is indicated. (F) Nanoparticle tracking analysis. Representative analyses of number (left) and average size (right) of particles recovered by ultracentrifugation from WT and null conditioned medium (median signal from 10 technical replicates). Inset graphs show quantification of each (mean ± SD, *n* = 3 biological replicates).

Our physical characterization of shed VSG indicates that most VSG is released directly from the cell surface in WT cells. However, small amounts of VSG are shed in EVs, and this makes a minor contribution to VSG turnover in WT and null cells. To assess whether loss of GPI-PLC affects EV production, we performed scanning electron microscopy (SEM) to quantify cell-associated nanotubes, which are the precursors of free EVs (28). Both WT and null cells had the rugose surface topology typically seen at low voltages (33) (Fig. 4 A). Cells bearing nanotubes (∼10%) (Fig. 4B) were quantified for number, length, and diameter (Fig. 4C to E). The only noticeable difference in nanotube production was a small but statistically significant increase in the diameter in null cells (132.6 ± 34.0 nm versus 108.0 ± 30.1 nm, *P* = 0.013). To further investigate this phenomenon, EVs were purified from conditioned medium from cultures of WT and GPI-PLC^−/−^ cells (28) and analyzed by nanoparticle tracking. EVs from null cells were more abundant (Fig. 4F), consistent with the increased high-molecular-weight and detergent-sensitive material seen in BNG analysis. EVs from null cells were also larger in mean diameter (138.0 ± 2.6 nm versus 127.7 ± 1.5 nm, *n* = 3, *P* < 0.005), matching the increased diameter of cell-associated nanotubes. Unfractionated EVs in CM were confirmed to be VSG containing, and hence trypanosome derived, by immunofluorescent ImageStream analysis (see Fig. S3). These results indicate that shedding of VSG in EVs, although still a very minor component of total shed VSG, is increased in the absence of GPI-PLC.

10.1128/mBio.01725-21.3 FIG S3 ImageStream analysis of CM. WT (A), GPI-PLC null (B), and AnTat1.1 (C) CMs and HMI-9 medium (D) and PBS (E) controls were labeled with fluorescent anti-VSG221 IgG and analyzed by ImageStream flow cytometry. The regions of high fluorescence and side scatter signals corresponding to EVs are gated in yellow. (F) Quantification of each of these regions in each sample is presented. Trypanosome-derived VSG221-containing EVs are only found in WT and null cells. Download FIG S3, TIF file, 1.8 MB. Copyright © 2021 Garrison et al. 2021 Garrison et al. https://creativecommons.org/licenses/by/4.0/ This content is distributed under the terms of the Creative Commons Attribution 4.0 International license.

## DISCUSSION

It has long been thought that normal VSG turnover occurs by GPI-PLC-mediated hydrolysis of the GPI anchor, leading to slow release of soluble CRD^+^ VSG into the extracellular milieu (9). However, there is a topological problem with this model, as the cytosolically disposed GPI-PLC is sequestered from cell surface VSG in intact cells. GPI-PLC can cleave surface VSG when cells are disrupted by hypotonic or mechanical lysis (17, 18), but such unrestricted access would be catastrophic in intact cells. Nevertheless, GPI-PLC is found on the surface of differentiated, short stumpy bloodstream forms and actively releases VSG as a prelude to further differentiation when taken up by the tsetse (23). Thus, a model of slow regulated release by GPI hydrolysis cannot be excluded for normal VSG turnover in replicating BSF parasites. However, GPI-PLC null BSF cells are fully viable and able to differentiate into PCF with normal kinetics (34, 35), a process that requires replacement of the VSG surface coat with a new coat made of procyclins. In addition, null cells can also undergo VSG switching, indicating that GPI-PLC is not essential for antigenic variation (35). Thus, GPI-PLC activity is not required for other known processes involving turnover of VSG.

These issues, along with recent work demonstrating that bloodstream trypanosomes actively release EVs containing VSG (28), have led us to reinvestigate the relative extents to which GPI hydrolysis and EV formation contribute to VSG turnover. VSG is consistently lost from cells into the medium with a half-life of ∼26 h in WT cells, in good agreement with previous reports (9, 10). Furthermore, GPI-PLC null cells have significantly slower VSG turnover (*t*_1/2_ of ∼36 h), consistent with the hydrolysis model (9). Nevertheless, shedding continues in the null cells, albeit at reduced rate, formally proving that GPI-PLC is not required for normal VSG turnover. However, very little of this VSG is shed in a form that is consistent with inclusion in membranous EVs, i.e., susceptible to detergent treatment in floatation, gel filtration, or BNG analyses. Rather, it is shed directly from the cell surface as VSG homodimers with intact (CRD^−^) GPI anchors. The situation is more complicated in WT cells, where VSG is shed in approximately equal portions as GPI-intact and GPI-hydrolyzed (CRD^+^) forms. This finding indicates that normal VSG turnover is a bimodal process involving both direct dissociation from the cell surface and release by GPI hydrolysis. The latter accounts for the elevated rate of release from WT cells compared to that from the null cells. It is also likely that a hybrid process occurs in which one GPI anchor is hydrolyzed and the protein then dissociates with a single GPI anchor. In either case, the amount of VSG shed in detergent-sensitive EVs is a very small portion of the total, although loss of GPI-PLC does increase the size and abundance of these particles. Phosphatidylinositides are known to be GPI-PLC substrates (21, 22), and, presumably, this size effect is mediated by the disruption of phosphoinositide and/or diacylglycerol signaling pathways, as suggested in reference 20. The only other phenotype of GPI-PLC knockout is the appearance of a VSG fragment in both cell and medium fractions of GPI-PLC-deficient cells, which is reminiscent of the proteolytic cleavage that takes place during differentiation to procyclics (23). This may represent dysregulation of the metalloprotease MSP-B that is upregulated for VSG release during differentiation (30). Whatever the explanation, the phenotype is a real consequence of GPI-PLC deficiency, as the addback completely eliminates generation of the fragment.

Our findings raise two issues. First is the GPI-PLC topology problem: the enzyme is on the cytoplasmic side, while the substrate is on the extracellular side. As mentioned above, active GPI-PLC is present on the surface of differentiated short stumpy parasites, but *a priori*, this seems risky in replicating long slender BSF parasites, where maintenance of an intact surface coat is paramount for survival. Consequently, we were initially doubtful about the original demonstration of VSG turnover by GPI hydrolysis (9). This concern was compounded by the two criteria used to evaluate the GPI status of released VSG. First, released material was judged to be hydrolyzed sVSG on the basis of electrophoretic mobility, but the difference in mobility between the two forms is subtle, requiring special electrophoretic methods such as urea or gradient gels to visualize (6, 17). Second, released VSG was found to be CRD^+^ by immunoblotting, but the relative amount of conversion was not assessed. Thus, it was possible that low-level GPI hydrolysis occurred but was mistaken for quantitative shedding of soluble VSG. However, our results demonstrate that GPI hydrolysis does account for approximately half of all VSG turnover. That said, it is likely this is an overestimate, as even a very low level of cell death during culture, which is impossible to avoid, would lead to GPI-PLC activation and VSG release. How GPI-PLC “translocates” to the cell surface, in either long slender or short stumpy parasites, is not clear. However, GPI-PLC is reversibly thioacylated (16), and it was suggested that this regulates access to cell surface VSG in intact cells (16, 23). Whatever the mechanism of translocation, it must be tightly regulated, as too much active surface GPI-PLC would likely be lethal.

The second issue is shedding of homodimeric VSG with two intact GPI anchors. This can only be by direct dissociation from the intact plasma membrane. We previously demonstrated that a single BSF GPI anchor is not sufficient to maintain membrane association (32). This is because the BSF GPI is composed exclusively of dimyristoylglycerol (36), which has a high off rate (*t*_1/2_ of ∼14 min) from biological membranes at 37°C (37, 38) and which is accentuated by attachment of bulky molecules, e.g., proteins. Furthermore, it has been observed that VSG can spontaneously transfer from live BSF trypanosomes into the membranes of nearby erythrocytes (39), a process that may contribute to the anemia associated with prolonged trypanosome infection. Apparently then, two dimyristoylglycerol GPI anchors are also insufficient to maintain membrane association, albeit with a much lower off rate.

These data along with those from previous studies on GPI-PLC function establish five principal functions for this enigmatic enzyme. First is normal turnover of VSG by GPI hydrolysis, in concert with direct shedding of intact VSG, in replicating BSF cells. Second, in partner with the metalloprotease MSP-B, is release of VSG from the cell surface during differentiation from short stumpy BSF to PCF stages. As noted above, GPI-PLC is present on the surface of short stumpy BSF parasites already releasing VSG prior to ingestion by the tsetse (23, 30). Release of soluble VSG into circulation by both replicating and short stumpy trypanosomes is likely to have significant effects on host innate immune responses (40). Third, GPI-PLC plays a poorly understood role in regulating receptor-mediated endocytosis (20). Fourth, GPI-PLC may be a metabolic enzyme. Although commonly thought to be GPI specific, it is fully active against PI (21). Furthermore, silencing in BSF cells show reduced incorporation of *myo*-inositol into inositol polyphosphates (22), suggesting that GPI-PLC cleaves higher-order phosphoinositides. Finally, during ER-associated degradation (ERAD) in BSF trypanosomes, misfolded GPI-anchored proteins are retrotranslocated from the ER lumen to the cytosolic side of the ER membrane, where they must be extracted before proteasomal degradation in the cytoplasm. We have shown that GPI hydrolysis precedes extraction and degradation, and the cytoplasmic disposition of GPI-PLC makes it the obvious candidate for this function (24).

In summary, we have reevaluated normal VSG turnover in actively dividing populations of BSF trypanosomes. As long thought, GPI hydrolysis is an important part of this process. However, direct shedding of VSG with intact GPI anchors may be the major mechanism and, in the absence of GPI-PLC, is capable of maintaining VSG turnover. In contrast, shedding of VSG in membrane-derived EVs plays a minimal role at best in VSG turnover. Thus, VSG turnover is essentially a bimodal process involving shedding of intact and GPI-hydrolyzed VSG homodimers.

## MATERIALS AND METHODS

### Cell lines and culture.

All work was carried out with cultured log-phase BSF cells of the commonly used tetracycline-responsive single-marker (SM221) Lister 427 strain of T. brucei *brucei* (variant antigenic type MITat1.2 expressing VSG221) (41), grown in HMI-9 medium (42).

### Hypotonic lysis assay.

Cells were washed with ice cold HEPES-buffered saline (HBS; 50 mM HEPES-KOH [pH 7.5], 5 mM KCl, 50 mM NaCl, 70 mM glucose) and suspended in ice-cold distilled water (dH_2_O) (10^7^ cells, 180 μl) with protease inhibitor cocktail (PIC; 2 μg/ml of leupeptin, antipain, chymostatin, pepstatin A, and aprotinin) and *N*_α_-tosyl-l-lysine chloromethyl ketone hydrochloride (TLCK; 0.37 μg/ml). Samples were kept on ice for 5 min and then supplemented with 20 μl of 10× TEN buffer (1×: 50 mM Tris-HCl, pH 7.5, 150 mM NaCl, 5 mM EDTA) and incubated at 37°C for 10 min to activate endogenous GPI-PLC. Membrane and supernatant fractions were prepared by centrifugation at 4°C. Samples were immunoblotted simultaneously with specific anti-VSG and anti-BiP antibodies.

### VSG turnover assay.

BSF trypanosomes were radiolabeled (15 min) with [^35^S]methionine/cysteine as described in reference 43 using methionine/cysteine-free TM-B medium (44), washed in HBS, and resuspended in prewarmed (37°C) HMI-9 growth medium at 5 × 10^4^ cells/ml. Incubation was continued for 24 h. At each time point, two consecutive volumes of cells (1 ml total) were gently centrifuged (3,000 × *g*, 1 min) in 0.45-μm SpinX centrifuge filters (Corning Inc., Salt Lake City, UT). Pass-through medium samples were supplemented with one-tenth volume of 10× radioimmunoprecipitation assay (RIPA) detergents (final, 1% NP-40, 0.5% deoxycholate, 0.1% SDS) and reserved on ice. The filter inserts with cells were transferred to fresh tubes, and cells were lysed with two consecutive volumes of 1× RIPA buffer (1 ml total), again with gentle centrifugation. All medium and lysate samples were then subjected to standard immunoprecipitation.

### Conditioned medium floatation assays.

To generate VSG-containing conditioned medium (CM), washed cells were resuspended at 10^7^/ml in HMI-9 medium and incubated for 6 h at 37°C. Following incubation, cells were removed on Costar SpinX centrifuge filters (0.45 μm; Corning Inc., Salt Lake City, UT), and the cleared CM was supplemented with protease inhibitor cocktail (PIC; 2 μg/ml of leupeptin, antipain, chymostatin, pepstatin A, and aprotinin). For analytical step floatation gradients, CM was adjusted to 40% OptiPrep (Sigma-Aldrich, St. Louis, MO), and 350 μl was overlaid with 35% OptiPrep phosphate-buffered saline (PBS) (600 μl) and 10% OptiPrep-PBS (450 μl) in thick wall polycarbonate TLS-55 tubes (Beckman Coulter Inc., Brea, CA). After centrifugation (TLS-55 rotor, 200,000 × *g*, 2 h, 4°C), gradients were manually fractionated from the top (5 × 280-μl fractions). Samples were fractionated by SDS-PAGE and immunoblotted with anti-VSG221 VSG or anti-CRD antibodies (12).

### Size exclusion chromatography.

qEVoriginal/35-nm columns (EV35; iZon, Medford, MA) were washed with PBS and then loaded with CM (0.5 ml). The column was eluted with PBS, and 0.5-ml fractions were collected. The *A*_280_ for each fraction was measured by NanoDrop spectrophotometry. Individual fractions were subjected to SDS-PAGE and immunoblotted with an anti-VSG221 antibody. For detergent treatment, CHAPS was added to load fractions (16 mM final; 2× CMC), and the column was equilibrated and run in PBS with 1 mM CHAPS.

### Scanning electron microscopy.

SEM was performed as described previously, and cells were fixed and dehydrated as described previously (33). Briefly, late-log-phase cells (0.5 × 10^5^ to 1.0 × 10^5^/ml) were fixed with 2.5% EM-grade glutaraldehyde (Electron Microscopy Sciences) in complete HMI-9 medium (2 h at 4°C). Cells were then gently collected by syringe-passage onto 0.2-μm-pore polycarbonate filters (Whatman Nuclepore, 25-mm diameter; Sigma-Aldrich) keeping fluid in the upper filter chamber (Whatman Swin-Lok cartridge, 25 mm; Sigma-Aldrich) in all subsequent steps until final air drying. Cells were washed and dehydrated through an ethanol series and finally dried with hexamethyldisilazane. Filters were removed and air dried, and samples were coated with evaporated carbon at high vacuum (Denton 502 evaporator). Images were acquired on a Hitachi SU70 field emission scanning electron microscope (FESEM) at 2.0 keV using an in-lens secondary electron detector at zero tilt or a lower detector at 70° tilt. Nanotubes were quantified from 3 biological replicates of 100 randomly selected cells in zero-tilt images.

### Nanoparticle tracking analysis of EVs.

EVs were purified as described in reference 28 from conditioned medium prepared from WT and GPI-PLC null cells. In brief, cells were grown overnight in complete HMI-9 medium with EV-depleted fetal bovine serum (FBS). Cells were removed by centrifugation at 3,000 × *g* for 15 min, followed by an additional spin at 10,000 × *g* for 15 min to remove large debris, and lastly filtered through a 0.45-μm filter to create conditioned medium. EVs were pelleted by ultracentrifugation in a Ti45 rotor at 120,000 × *g* for 1 h 34 min and washed twice with PBS. The concentration, diameter, and labeling intensity of ultracentrifugation-purified EVs were determined using the NanoSight NS300 (Malvern Panalytical, Malvern, UK). For each sample, 10 replicate videos were captured for 1 min each using a 532-nm laser at camera level of 14. Analysis of videos was performed within NanoSight NTA software 3.2 Dev Build 3.2.16 using the settings of detect threshold 4, auto blur size, and auto max jump distance.

### Imaging flow cytometry.

Protein A-purified rabbit VSG221 antibody was conjugated with Alexa Fluor 555 (Invitrogen, catalog no. A37571) and the degree of labeling was determined to be 1.8 molecules per antibody. Antibody solution was precleared to remove aggregates by spinning at 16,000 × *g* for 30 min at 4°C. EVs were labeled with 25 mg/ml (20 ml final volume in PBS) of labeled antibody for 30 min in the dark at room temperature and then diluted 5-fold and placed on ice before being processed. Imaging flow cytometry acquisition was performed on an ImageStream^X^ Mk II (Luminex Corporation, Seattle, WA) with fluidics set at medium speed, sensitivity set to high, magnification set to 60×, and laser power set to high. All samples were acquired using the autosampler with INSPIRE software for an acquisition time of 2 min per sample. Three or four technical replicates were performed for all samples, with a PBS wash in between each sample type. Gates were set and quantified using IDEAS software version 6.2. *P* values shown were acquired from GraphPad Prism v7, with each row analyzed individually and a two-stage step-up method as described in reference 45.

### Data analyses.

ImageJ (http://imagej.nih.gov/ij/) was used to quantify data from phosphorimager assays and fluorescence blot scans. For quantitative analysis of band intensities, signals from each lane were subtracted from the signal of the equivalent unlabeled areas of that lane. In the case of the VSG turnover assay, the zero supernatant signal was subtracted from all subsequent supernatant signals. Data analyses were performed with Prism 6 software (GraphPad Software, Inc., San Diego, CA). Biological replicates (*n*) were obtained as indicated in the text.
