# Supplementary figures and images for: Turnover of Variant Surface Glycoprotein in Trypanosoma brucei Is a Bimodal Process
Source: mBio. 2021 Jul 27;12(4):e01725-21. doi: 10.1128/mBio.01725-21 (PMC8406259; doi:10.1128/mBio.01725-21)

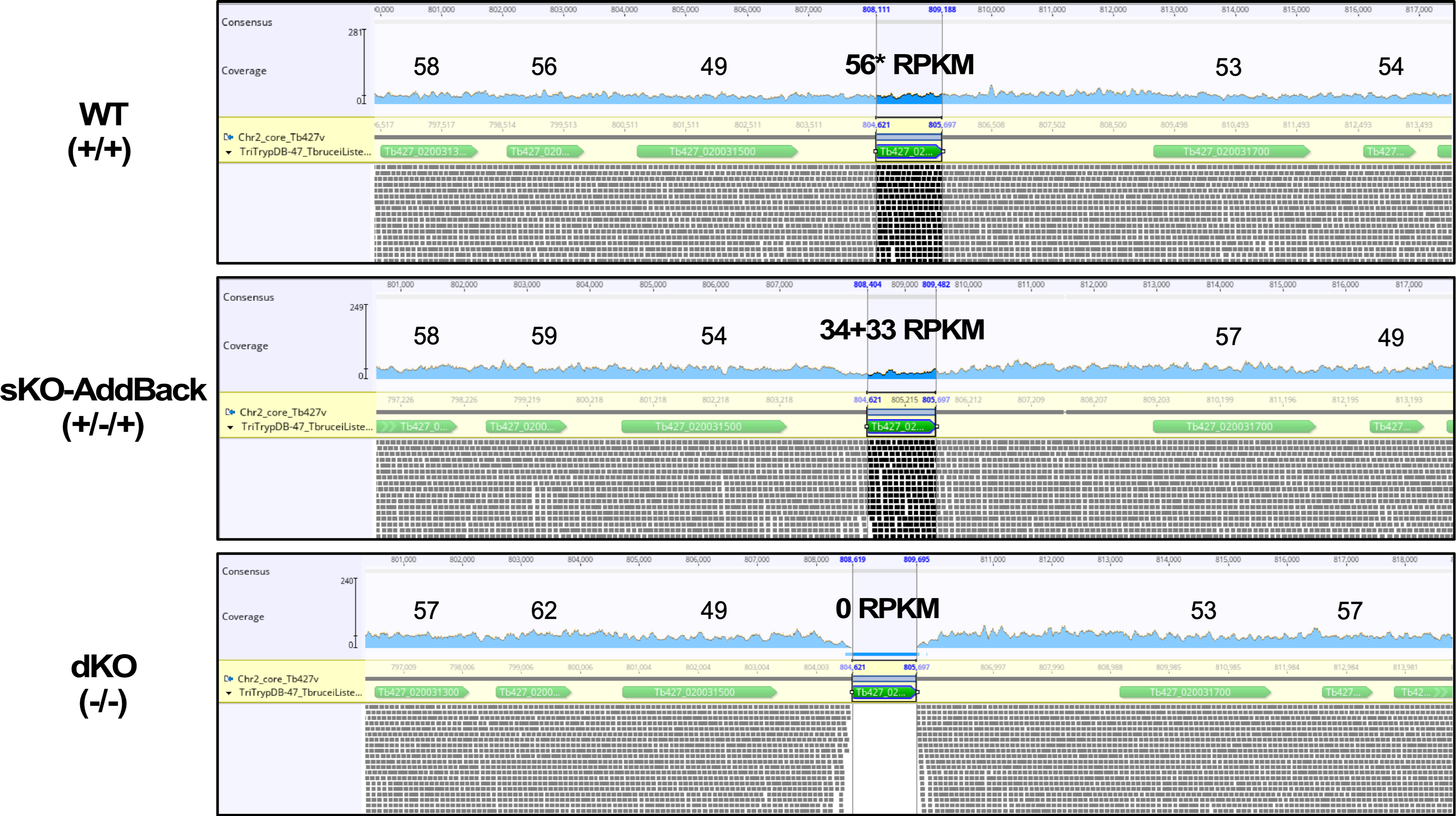

Supplement: FIG S1 [file mbio.01725-21-sf001.tif]

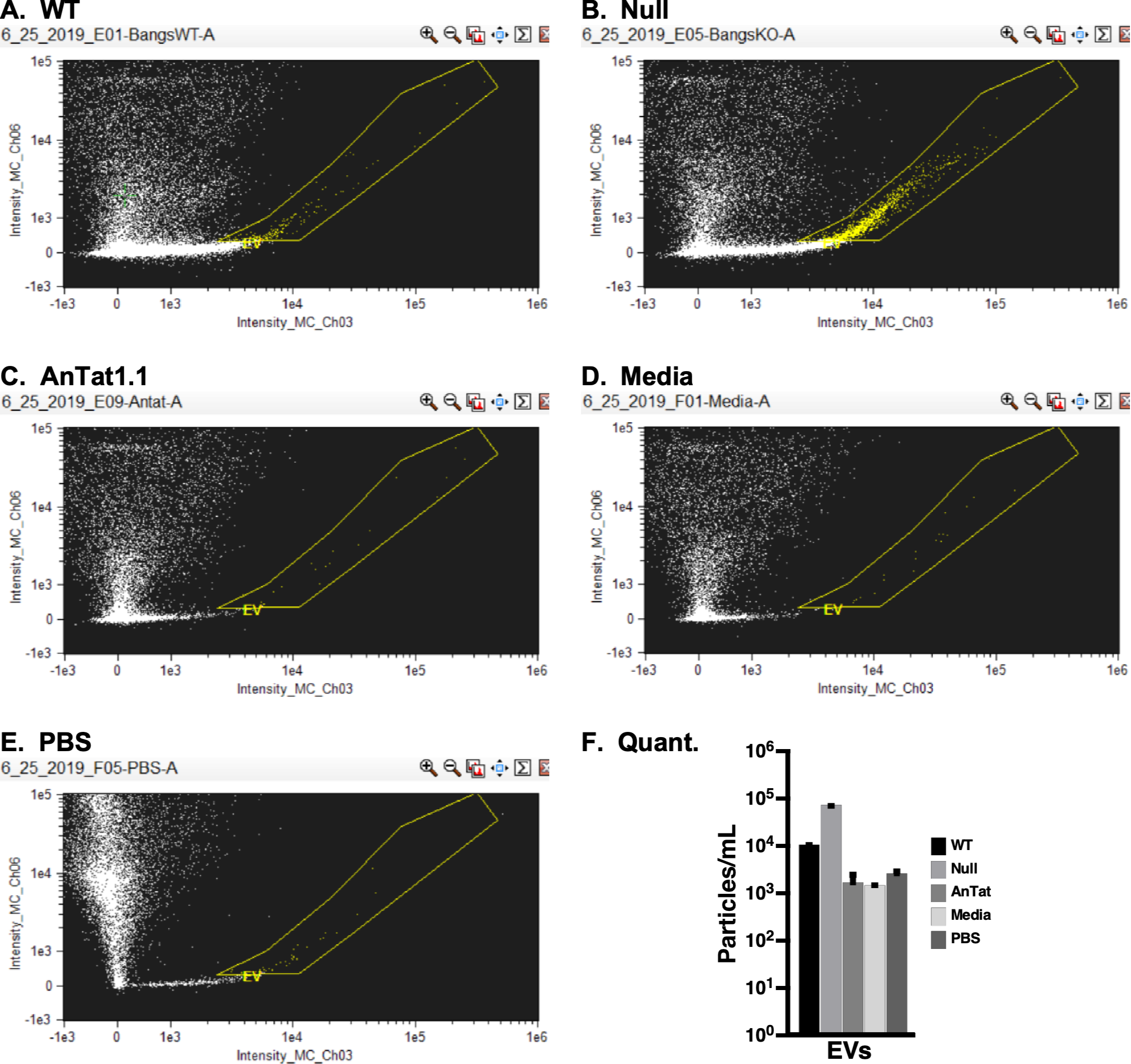

Supplement: FIG S3 [file mbio.01725-21-sf003.tif]
